# INVect - a novel polycationic reagent for transient transfection of mammalian cells

**DOI:** 10.1186/1753-6561-7-S6-P26

**Published:** 2013-12-04

**Authors:** Sebastian Püngel, Miklos Veiczi, Tim Welsink, Daniel Faust, Vanessa Vater, Derek Levison, Uwe Möller, Wolfgang Weglöhner

**Affiliations:** 1 InVivo BioTech Services GmbH, 16761 Hennigsdorf, Germany; 2 emp Biotech GmbH, 13125 Berlin, Germany

## Background

For rapid recombinant protein production in small to medium size volumes, transient transfection of mammalian cells is still the method of choice in biotechnology [1]. However, due to the high costs of commercially available lipofectamines or polycationic transfection reagents such as polyethylenimine (PEI), the most widely used transfection reagents available present a substantial economic bottleneck. While these reagents produce seemingly high transient transfection rates [2], there is still a strong desire for transfection reagents providing both secure and easy handling and higher recombinant protein production. As part of our commitment to excellence, InVivo BioTech Services initiated a joint venture with emp Biotech and developed a novel polycationic reagent, named INVect, for transient transfection and recombinant protein production in mammalian cells.

## Materials and methods

Mammalian cells were cultured in CD-ACF media using shake flasks and standard culture conditions. Cells were transfected with 10 μg per mL of a GOI harboring plasmid at a cell density of 5 × 10^6 ^cells per mL in FreeStyle™ Medium (Life Technologies) with INVect to DNA ratio of 6:1 (w/w) and PEI to DNA ration of 2:1 (w/w). Cultures were supplemented with same volume Protein Expression Medium (Life Technologies) 2 hours post transfection. GFP and SEAP expression took place in 8 mL culture volume in 50 mL bioreactor tubes. Expression of other reporter proteins were performed in 150 mL culture volume in 500 mL shake flasks. Transfection efficiency was determined 24 hours post transfection by counting green fluorescent positive cells using a FACSCalibur (Becton, Dickinson and Company). SEAP expression was determined in cell culture supernatant on day 6 post transfection by a photometric pNPP turn-over assay. Quantification of IgG was performed by protein G affinity chromatography on day 6 post transfection. Thrombomodulin concentration was calculated from cell culture supernatant on day 6 post transfection by IMUBIND^® ^Thrombomodulin ELISA Kit (american diagnostica). His-tagged recombinant protein was purified on day 6 post transfection by TALON^® ^immobilized metal affinity chromatography system.

## Results

**Figure 1:**
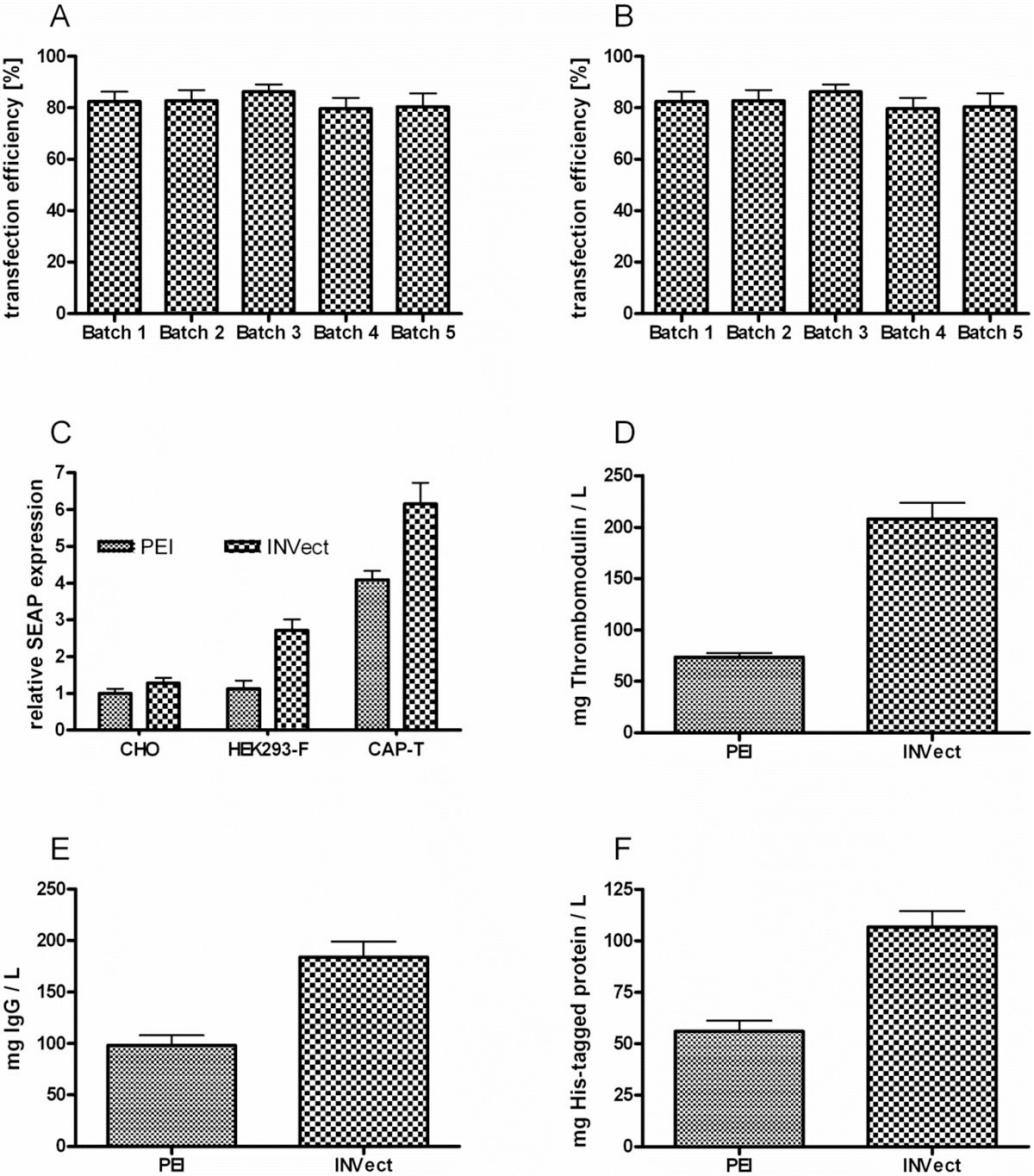
**Transfection efficiency and 6 day post-transfection cell productivity of INVect**. (A) Transfection efficiency of INVect compared to PEI. (B) transfection efficiency of 5 independent batches. Transfection efficiency was determined 24 hours post transfection by counting green fluorescent positive CAP-T cells using a FACSCalibur (Becton, Dickinson and Company). (C) CHO-S, HEK293-F and CAP-T cells were transfected with a *SEAP *harboring plasmid. Relative SEAP expression was determined in cell culture supernatant by a photometric pNPP turn-over assay. (D) CAP-T cells were transfected with a *Thrombomodulin *harboring plasmid. Thrombomodulin concentration was calculated from cell culture supernatant by IMUBIND^® ^Thrombomodulin ELISA Kit (american diagnostica). (E) CAP-T cells were transfected with an *IgG *harboring plasmid. Antibody concentration was determined by protein G affinity chromatography. (F) CAP-T cells were transfected with a *His-tagged protein *harboring plasmid. Protein of interest was purified by TALON^® ^immobilized metal affinity chromatography system.

Cytotoxicity was tested over a broad range of concentrations. Results demonstrate several novel synthetic polymers exhibiting transfection efficiencies even higher than common PEIs after optimized ratios of DNA-to-polymer were applied. Transfection efficiency of INVect was compared to PEI, currently the standard transfection reagent for transient gene expression. INVect was found to generally give better transfection efficiencies of greater 80% in a GFP assay (Figure 1A). Batch-to-batch reproducibility was shown on five independent INVect batches. Transfection results were highly consistent and in the range of 80-90% (Figure 1B).

INVect successfully delivers genes to HEK293-F, CHO-S and CAP-T cells as shown in a SEAP expression system (Figure 1C). Post-transfection cell productivity was determined under TGE manufacturing conditions. Thrombomodulin (60 kDa) (Figure 1D), an IgG (144 kDa) (Figure 1E) and a HIS-tagged Protein of Interest (~40 kDa) (Figure 1F) were transiently expressed using INVect as transfection reagent and conventional 25 kDa PEI as control. Cells were transfected with a gene of interest harboring plasmid, with product concentration being measured on day 6 post transfection. The use of INVect provided a minimum 2-fold increase in protein production over PEI (25 kDa) based transfection.

## Conclusions

INVect is a novel polycationic transfection reagent which demonstrates low cell toxicity for transient transfection of mammalian cells and delivers extremely high transfection efficiencies of up to 90%, 24 h post transfection. The use of INVect for transfection under TGE conditions leads to exceptionally high levels of protein expression and outperforms 25 kDa linear PEI by 2-fold. INVect can be used effectively with all common cell lines and is especially suited for HEK293-F and CAP-T cells.
